# Hippocampal mismatch signals are based on episodic memories and not schematic knowledge

**DOI:** 10.1073/pnas.2503535122

**Published:** 2025-08-22

**Authors:** Dominika K. Varga, Petar P. Raykov, Elizabeth Jefferies, Aya Ben-Yakov, Chris M. Bird

**Affiliations:** ^a^School of Psychology, University of Sussex, Brighton BN1 9QH, United Kingdom; ^b^Sussex Neuroscience, University of Sussex, Brighton BN1 9QH, United Kingdom; ^c^Medical Research Council Cognition and Brain Sciences Unit, University of Cambridge, Cambridge CB2 7EF, United Kingdom; ^d^Department of Psychology, University of York, York YO10 5DD, United Kingdom; ^e^Edmond and Lily Safra Center for Brain Sciences, Hebrew University of Jerusalem, Jerusalem 9190401, Israel

**Keywords:** hippocampus, prediction, mismatch, episodic memory, schema knowledge

## Abstract

Prediction errors drive learning by signaling mismatches between expectations and reality, but the neural systems supporting these computations remain debated. The hippocampus is implicated in mismatch detection, yet it is not known whether it signals mismatches with episodic memories or generalized knowledge. Across three functional Magnetic Resonance Imaging (fMRI) experiments, we show that the hippocampus selectively responds to mismatches with episodic memories of specific events. In contrast, schematic mismatches engage Semantic Control and Multiple Demand Networks, as well as subcortical regions linked to prediction error signaling. Episodic mismatches also recruit the Default Mode Network. These findings challenge accounts that propose the hippocampus is a domain-general mismatch detector. Instead, the findings support a more specialized role for the hippocampus in learning that is underpinned by its well-established importance in processing episodic memories.

## Significance

Our brains use memories of the past to make sense of the present and predict the future. These memories might be of specific events or more general knowledge about the world. The hippocampus is widely implicated in signaling mismatches with memory-based predictions, but whether it uses specific episodic memories or generalized knowledge remains unclear. We show that the hippocampus selectively signals mismatches with episodic memories, while other brain networks respond to unexpected situations more broadly, regardless of memory type. These findings clarify the hippocampus’ role as a comparator, showing it is specialized for evaluating reality against episodic memories, and offer insight into how the brain uses past experiences to interpret the present and anticipate the future, shaping learning and memory.

Humans possess a remarkable ability to predict what will happen in new situations based on past experiences (1–3). Detecting a mismatch between our predictions and our in-the-moment experience offers a powerful route to rapidly learn new information (e.g., refs. 4 and 5). The hippocampus plays a central role in mismatch detection (e.g., refs. 6–11). However, it is not known whether the hippocampus computes mismatches between current experience and predictions based on general schematic knowledge about the past or episodic memories of specific earlier experiences.

Several proposals suggest that the hippocampus detects novelty by comparing incoming information with stored representations. This includes processing associative mismatch novelty [e.g., where familiar objects are reconfigured into novel arrangements (e.g., ref. 12)], contextual novelty [where items are unexpected within a given context (e.g., ref. 13)], and schema incongruence [violations of structured knowledge about the world (e.g., ref. 14)]. These proposals are based on the well-established specialization of the hippocampus for processing relations between items and particularly item-context associations (15–19). Furthermore, the neural circuitry within the hippocampus is particularly suited to its hypothesized role as a comparator (9, 20). However, the prior contextual representations used in this comparator function remain unclear.

There is strong evidence that the hippocampus supports mismatch processing based on specific past experiences to compare to current situations. In humans, it has been shown that the hippocampus detects changes in recently learned cue-outcome associations (e.g., refs. 21–23) and sequences of events (e.g., refs. 24–26). Likewise, in animal studies, the hippocampus shows a mismatch signal when changes are made to specific, previously encountered environments (27–29). Some computational models propose that the comparator function of the hippocampus is limited to processing mismatches with episodic-like representations of specific events (7, 30).

However, often our expectations are based on our generalized understanding of patterns and regularities developed across multiple similar experiences (31–33). It has been suggested that the hippocampus may also process mismatches based on these more generalized representations (34–36). This argument is based on evidence that the hippocampus learns the common elements and temporal regularities across multiple past experiences (37–39), can infer relationships between items that have never been directly experienced together (40), and is involved in imagining complex future scenarios (41). Some computational models have proposed that the hippocampus plays a key high-level role within a “generative model” that makes predictions about the state of the world based on abstract generalized knowledge (10, 42). However, it remains unclear whether the hippocampus uses these generalized knowledge structures to compare expectations with reality.

**Fig. 1.**
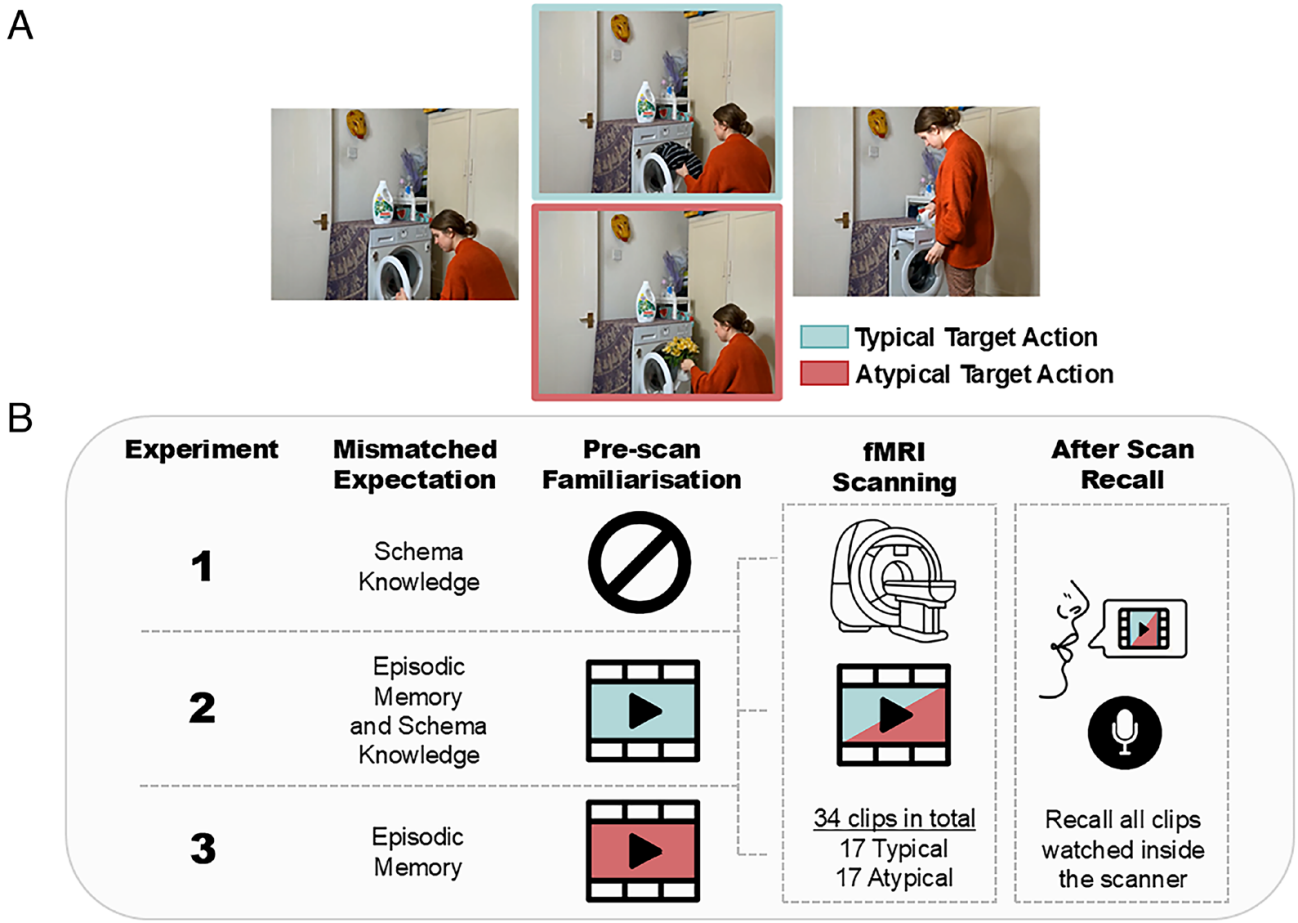
Experimental paradigm. (*A*) Still frames showing example moments from the two alternative versions of the “laundry” video clip (watch the clips here: https://e01.eventmemory.org/ExampleVid_paper.html). The two versions showed a nearly identical sequence of actions, except for the target action that was either Typical or Atypical. (*B*) Before scanning, participants either did not watch any clips (Experiment 1), watched the Typical version of each clip (Experiment 2), or watched the Atypical version of each clip (Experiment 3). Across three experiments, all participants watched half of the clips in the Typical and the other in the Atypical version during fMRI. By manipulating prescan familiarity with the clips, target actions in each experiment violated different types of expectations. After scanning, participants were asked to describe what happened in all video clips watched inside the scanner, focusing on the actions the actors performed, cued by the first frame of each clip.

The critical test of all these theories of hippocampal mismatch detection is whether the hippocampus responds to mismatches with generalized knowledge or whether its role is limited to processing comparisons based on episodic-like memories. We address this using three functional Magnetic Resonance Imaging (fMRI) experiments, where we manipulated the source of prior knowledge (a form of expectation) while participants watched custom-made video clips of actors performing sequences of everyday actions (e.g., doing the laundry). Inside the scanner, all participants watched half of the clips in their “Typical” version (e.g., putting clothes into a washing machine) and the other half in their “Atypical” version (e.g., putting flowers into the washing machine). Depending on participant’s prescan familiarity with the clips, actions in the clips mismatched different types of expectations. When participants were unfamiliar with the clips prior to scanning, Atypical actions mismatched solely general Schema Knowledge (Experiment 1). When participants had prewatched all clips in their Typical version, Atypical actions mismatched both Schema Knowledge and Episodic Memory of the specific clips (Experiment 2). Finally, when participants had prewatched all clips in their Atypical version, Typical actions mismatched Episodic Memory only (Experiment 3) (Fig. 1).

In addition to our primary hippocampal analyses, to more comprehensively characterize the neural systems involved in processing unexpected events, we conducted exploratory whole-brain analyses as well as region of interests (ROI) analyses in the Default Mode Network (DMN), Semantic Control Network (SCN), and Multiple Demand Network (MDN). These networks were selected based on their differing roles in processing ongoing experiences. The DMN has been implicated in using past episodic information to support the interpretation of situations as they unfold across time (e.g., refs. 43 and 44). The SCN supports retrieval and integration of semantic knowledge in contextually appropriate ways, particularly when incoming information is unexpected or ambiguous (45). Meanwhile, the MDN is known to provide domain-general attentional resources in response to surprising or difficult-to-interpret stimuli (46). By examining responses across these regions, we aimed to better understand the broader network dynamics that support the processing of different types of unexpected events.

We also included the ventral tegmental area and substantia nigra (VTA/SN) as an exploratory ROI based on their established role in processing prediction errors and their functional connections with the hippocampus. The hippocampus is thought to signal unexpected events to midbrain dopaminergic regions, which then modulate memory encoding (9). These midbrain structures are known to signal motivational salience and update expectations when predictive relationships are violated.

Overall, we found strong evidence that the hippocampus is limited to using episodic memory-based representations for its comparator mechanism. Responses to schematic knowledge-based mismatches were found in regions outside of the hippocampus, including cortical control networks and subcortical regions implicated in prediction error processing.

## Results

### The Effect of Expectation Violation on Memory.

**Fig. 2.**
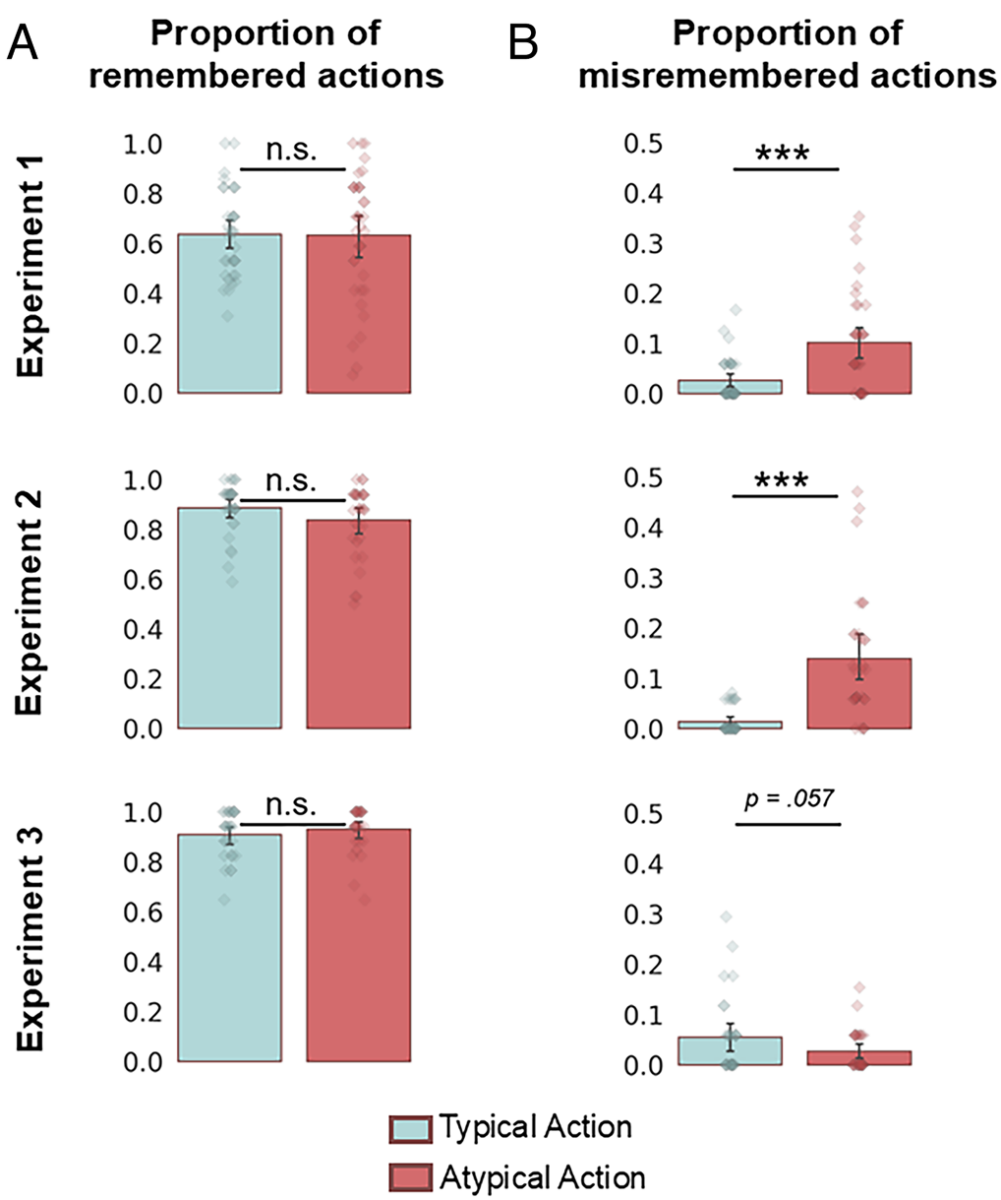
Memory was influenced by the expectedness of actions. (*A*) Bar charts showing the average proportion of remembered target actions in the Typical and Atypical conditions in each experiment. Error Bars represent 95% CI. Strip plots show each participant’s average proportion of remembered target actions for each condition. There were no differences in the average proportion of correctly recalled target actions between conditions. (*B*) Bar charts showing the proportion of erroneously recalled target actions in each condition. People made more errors when recalling actions that violated their expectations. Error Bars represent 95% CI. ***P < 0.001, n.s. *P* > 0.05.

After scanning, participants were asked to recall the sequence of actions depicted in each video clip that they watched inside the scanner. We analyzed memory for the target actions specifically, showing high recall accuracy across all experiments (Fig. 2*A*). It is notable that participants’ accuracy significantly increased across the three experiments [β = 1.23; 95% CI = [1.09 1.37]; Z = 16.85; *P* < 0.001; Correct Recall ~ Experiment + (1 | Participant) + (1 | Video Clip)], likely reflecting participants’ increased familiarity with the videos in Experiments 2 and 3 due to the additional prescanning phases in these experiments. Interestingly, despite these differences in familiarity, the within-experiment logistic mixed-effects models [Correct Recall ~ Condition + (1 | Participant) + (1 | Video Clip)] showed no significant differences in recall accuracy between Typical vs. Atypical actions in any experiment (Experiment 1: β = 0.12; 95% CI = [−0.57 0.81]; Z = 0.33; *P* = 0.739; Experiment 2: β = −0.59; 95% CI = [−1.23 0.06]; Z = −1.79; *P* = 0.074; Experiment 3: β = 0.42; 95% CI = [−0.31 1.15]; Z = 1.14; *P* = 0.254).

Next, we ran further exploratory analyses to test whether participants were more likely to *misremember* the target actions, depending on whether the action violated expectations (Fig. 2*B*). Actions were counted misremembered if the correct action was mentioned, but the object was unspecified or replaced with another object. Participants made considerably more errors when recalling the Atypical compared to the Typical actions in Experiments 1 and 2 (Experiment 1: β = 1.51; 95% CI = [0.77 2.25]; Z = 3.99; *P* < 0.001; Experiment 2: β = 2.48; 95% CI = [1.61 3.34]; Z = 5.64; *P* < 0.001). There was only a marginally significant difference in the proportion of memory errors between conditions in Experiment 3 [β = −0.81; 95% CI = [1.61 3.34]; Z = −1.91; *P* = 0.057; (Memory Error Score ~ Condition + (1 | Participant) + (1 | Video Clip))]. Interestingly, the effect in Experiment 3 was in the reverse direction to the effects in Experiments 1 and 2; participants were numerically more likely to misremember the Typical actions, having prewatched versions of the videos showing the Atypical actions. Overall, across all experiments, participants were more likely to misremember unexpected target actions, either by replacing the object involved (e.g., “she put fruits into the washing machine”) or recalling the action without specifying the object (e.g., “she put something strange into the washing machine”).

In summary, participants remembered the target actions well, and the actions were remembered differently depending on their expectedness. Importantly, there was no difference in correctly recalling actions that violated or met contextual expectations. However, participants were more likely to make errors when recalling unexpected actions.

### The Effect of Different Types of Expectation Violation on Hippocampal Activity.

**Fig. 3.**
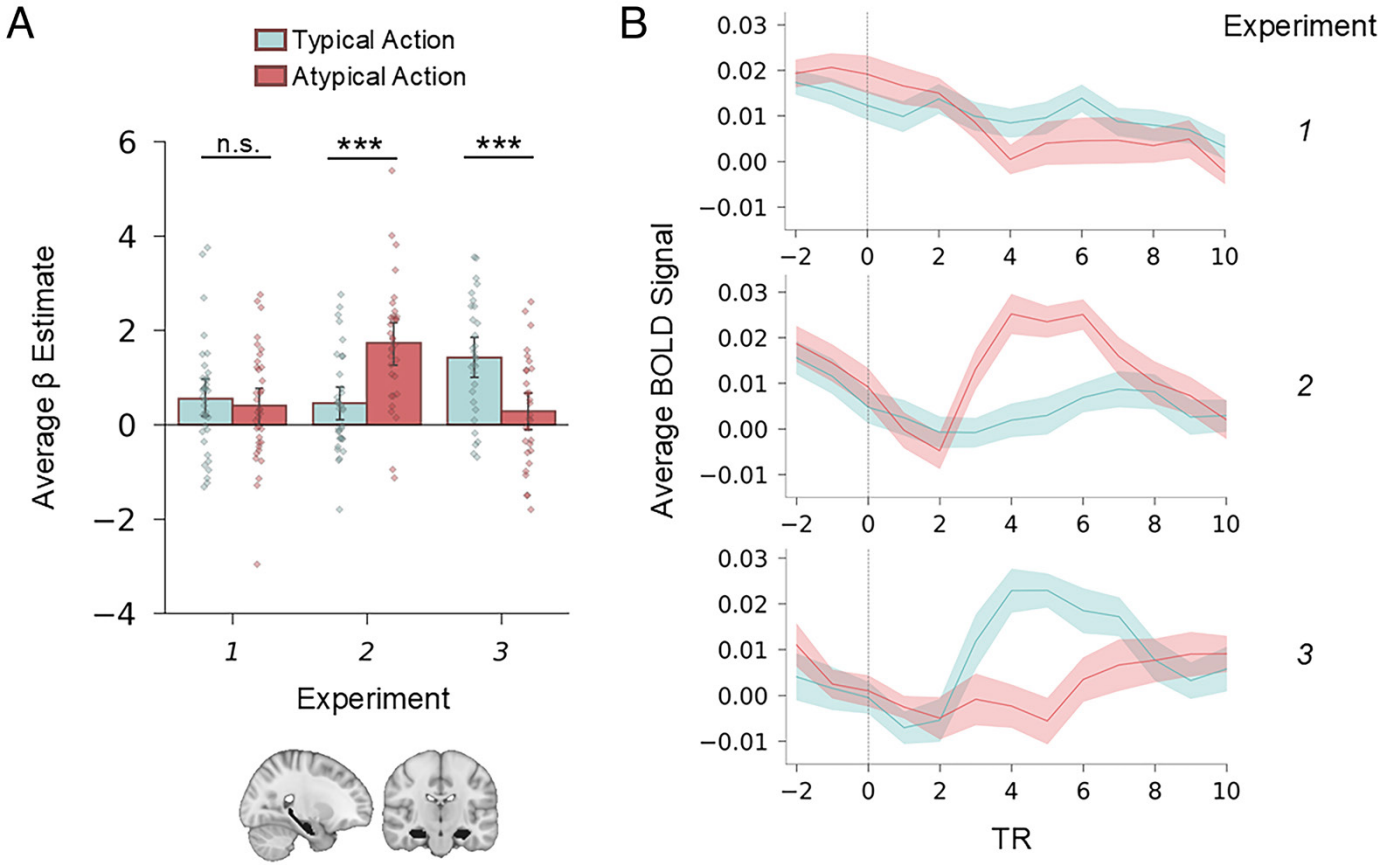
Hippocampal mismatch response is limited to signaling episodic memory–based expectation violation. In Experiment 1, expectations are based only on schematic knowledge, in Experiment 2, expectations are based on both episodic memory and schematic knowledge, and in Experiment 3, expectations are based only on episodic memory. (*A*) Bar charts showing the average response to Typical and Atypical target actions in the bilateral hippocampus in the three experiments (strip plots show individual participants’ averaged parameter estimates for the target actions estimated from a GLM). Error bars represent 95% CIs. (*B*) Time course of average BOLD signal in the bilateral hippocampal ROI to Typical and Atypical target actions in each experiment. ***P < 0.001; n.s. *P* > 0.50.

Our main analyses focused on the hippocampus; the analyses and hypotheses of Experiments 1 (https://osf.io/7g82x) and 3 (https://osf.io/zbnt9) were preregistered; the analyses and hypotheses of Experiment 2 were not preregistered, but the analyses were identical to the other experiments. We aimed to test whether hippocampal activity is modulated by the expectedness of target actions under different types of prior expectations. We used a General Linear Model (GLM) to isolate transient activity evoked by the Typical and Atypical target actions. Hippocampal activity was quantified by averaging beta weights from all voxels within a bilateral hippocampal mask for each condition in each experiment, allowing us to directly compare responses to Atypical and Typical actions. The results are shown in Fig. 3.

In Experiment 1, our preregistered hypothesis was that hippocampal activity would be modulated by schema-based expectation violations. Specifically, we predicted that activity would be higher in response to Atypical compared to Typical actions in line with its proposed role as a general-purpose mismatch detector. However, a paired *t* test revealed that there was no effect of schema-based mismatches in the hippocampus (t_(35)_ = 0.57; with a mean difference of 0.14; 95% CI = [−0.36 0.65]; *P* = 0.567; and a negligible effect size of d = 0.12, 95% CI = [–0.30 0.54]), against our preregistered hypothesis. Bayesian analysis provided moderate evidence for the null hypothesis (BF_01_ = 4.78), further suggesting that hippocampal activity was not modulated by target action expectedness in this experiment. To examine whether adding specific episodic memory-based expectations while watching the same clips would elicit mismatch signals in the hippocampus, we conducted Experiment 2.

In Experiment 2, hippocampal activity was greater to Atypical compared to Typical actions (t_(32)_ = −4.59; with a mean difference of –1.28, 95% CI = [−1.84 −0.71]; *P* < 0.001; and a large effect size of *d* = –1.06, 95% CI = [–1.64 –0.48]; BF_10_ = 378.25). This reveals that being able to compare specific episodic memories to reality is important for eliciting a mismatch response in the hippocampus. However, the increased hippocampal response in Experiment 2 may reflect an additive effect of schema-based and episodic memory-based mismatches, leaving it unclear whether episodic memory violations alone would elicit increased activity.

To address this concern, we conducted Experiment 3, which tested solely episodic memory-based violations. Here, participants prewatched all the Atypical versions of the videos. Consequently, during scanning, the Typical versions of the videos were unexpected on the basis of episodic memory alone. Based on the results of Experiment 2, our preregistered hypothesis was that hippocampal activity would show greater response to the Typical compared to Atypical actions. In this experiment, hippocampal activity was significantly higher for Typical compared to Atypical actions (t_(29)_ = 5; with a mean difference of 1.13; 95% CI = [0.67 1.60]; *P* < 0.001; and a large effect size of *d* = 0.95, 95% CI = [0.49 1.40]; BF_10_ = 910.34), in line with the preregistered hypothesis. Overall, providing compelling evidence that the hippocampus responds to mismatches even when they are driven solely by episodic memory, thus ruling out the influence of schema-based expectations.

In supplementary analyses, we further tested whether the hippocampal response differed as a function of expectation type, conducting a direct comparison of the mismatch effect across Experiments 1 and 2 (*SI Appendix*, *Supplementary Results*). This analysis revealed a significant interaction between experiment and condition, confirming that the hippocampus responded more strongly to violations of specific episodic expectations than to violations of general schematic knowledge. We also report more fine-grained analyses on the effect of mismatches in hippocampal subregions (head, body, and tail) (*SI Appendix*, Fig. S2 and *Results, Hippocampal Subregions*) and subfields (CA1, subiculum, and a combined CA3/CA4/dentate gyrus region) (*SI Appendix*, Fig. S3 and *Results, Hippocampal Subfields*). These analyses all found consistent effects throughout the hippocampus.

### The Effect of Different Types of Expectation Violation Outside of the Hippocampus.

**Fig. 4.**
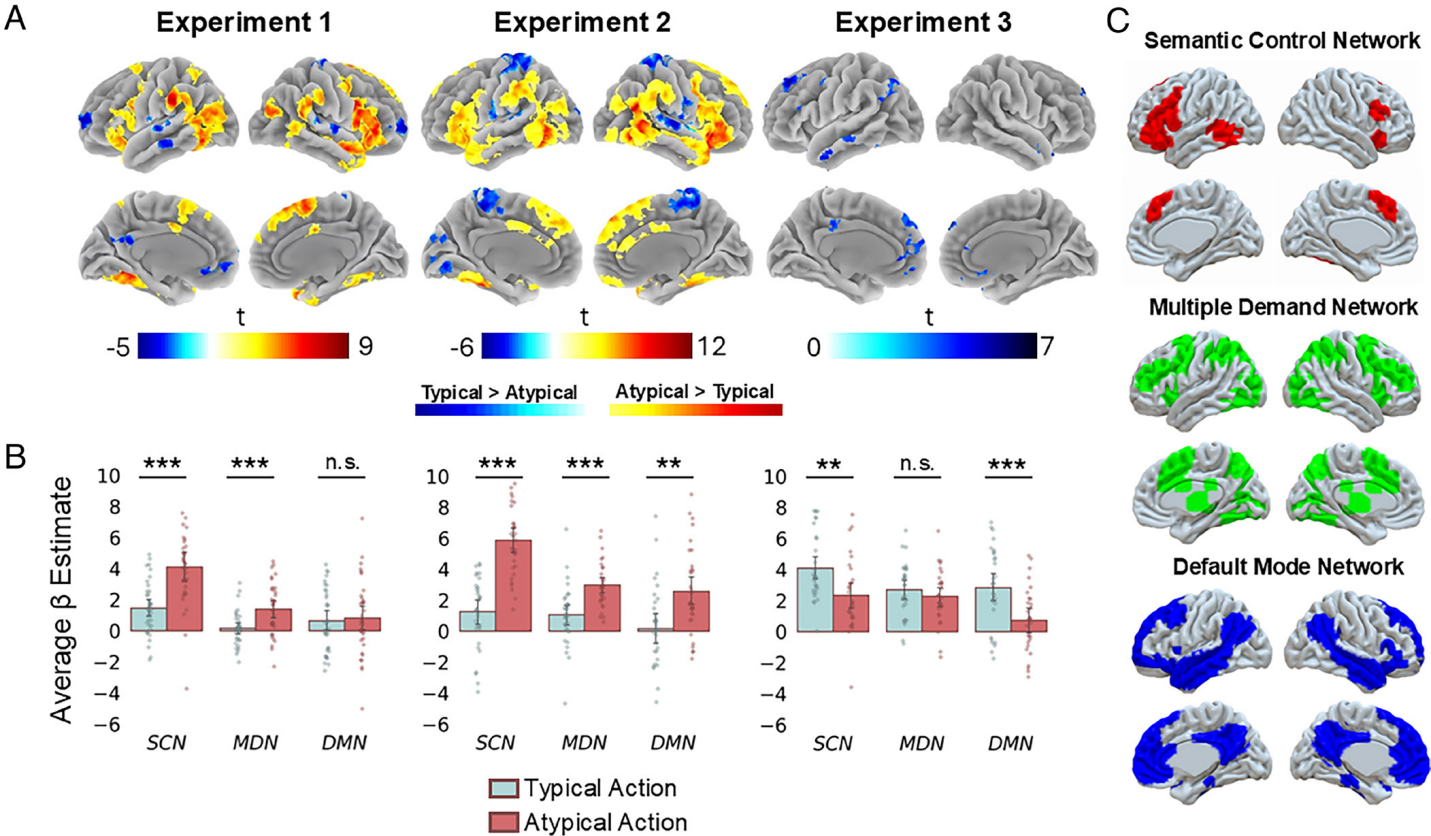
The effect of expectation across the whole brain and in three cortical networks. (*A*) T-maps of the contrast between Atypical and Typical target actions in each experiment. Whole-brain t-maps are cluster corrected at FWE *P* < 0.05 at voxel height defining threshold of *P* < 0.001 and color-coded to indicate the intensity of activation. The color bar indicates the t-statistic associated with each voxel. (*B*) Bar charts show the average response to Typical and Atypical target actions in the SCN, MDN, and DMN in the three experiments (strip plots show individual participants’ averaged parameter estimates for the target actions estimated from a GLM). Error bars represent 95% CIs. (*C*) Spatial maps of the network ROIs.

We conducted exploratory whole-brain analyses to investigate the mismatch responses outside of the hippocampus in all three experiments. First, we ran separate GLMs for the three experiments, modeling the response to the Atypical and Typical target actions. Group-level contrasts between the Atypical and Typical target actions are shown in Fig. 4*A*. We additionally carried out post hoc exploratory ROI analyses investigating whether the effects of expectation violation differed across three brain networks—the SCN, the MDN, and the DMN. Within each experiment, we conducted a repeated measures ANOVA between the target action conditions and Networks on the average parameter estimates associated with the target actions from all voxels comprising each network (Fig. 4*B*). A final exploratory ROI analysis focused on the ventral tegmental area (VTA)/SN.

First, in Experiment 1, Atypical compared to Typical actions engaged regions generally implicated in attentional engagement, semantic and predictive processing (47, 48). Mismatch signals were also present in the caudate nucleus (49), the amygdala (50), and the thalamus (51), consistent with previous work on expectation violations. Typical compared to Atypical actions engaged regions (such as Posterior Medial and Medial Prefrontal Cortex) implicated in encoding schema-consistent information, comprehending narratives, and mentalizing (52–54). This finding is in accordance with suggestions of the SLIMM model [schema-linked interactions between medial prefrontal and medial temporal regions (14)] that the medial prefrontal cortex has an important role in detecting the match between current events and existing contextual associations.

The network-level analysis revealed significant main effects for Condition (F_(1,35)_ = 13.70; *P* < 0.001; eta2[g] = 0.11) and Network (F_(2,70)_ = 35.60; *P* < 0.001; eta2[g] = 0.19), and a significant Condition*Network interaction (F_(1.65,57.7)_ = 14.07; *P* < 0.001; eta2[g] = 0.06). Pairwise comparisons showed that the average response to Atypical compared to Typical actions was significantly higher in the SCN (t_(35)_ = 5.64; *P* < 0.001) and MDN (t_(35)_ = 4.11; *P* < 0.001). Schema-based expectation violation did not significantly modulate activity in the DMN overall (t_(35)_ = 0.30; *P* = 0.763).

There was a significant increase in VTA/SN activity for the Atypical actions, consistent with this region responding to prediction errors (9, 55, 56). A paired *t* test comparing Atypical (M = 0.71, SD = 1.53) and Typical (M = −0.30; SD = 1.22) actions revealed an effect of condition (t_(35)_ = 2.90, *P* = 0.006, 95% CI = [−1.72 −0.30]).

In Experiment 2, a strikingly similar map of regions was activated more by Atypical than Typical actions (Fig. 4). Once again, subcortical effects were present in the caudate nucleus, thalamus, and amygdala. The Network ANOVA revealed significant main effects for Condition (F_(1,32)_ = 57.76; *P* < 0.001; eta2[g] = 0.30) and Network (F_(2,64)_ = 24.87; *P* < 0.001; eta2[g] = 0.14), and a significant Condition*Network interaction (F_(1.29,41.26)_ = 11.19; *P* < 0.001; eta2[g] = 0.06). Pairwise comparisons showed that the average response to Atypical target actions was again significantly higher in regions of the SCN (t_(33)_ = 10.1; *P* < 0.001) and MDN (t_(33)_ = 4.95; *P* < 0.001). Now with the addition of context-specific memory-based predictions, regions of the DMN were also overall more engaged by Atypical than Typical actions (t_(33)_ = 3.52; *P* = 0.001).

In the VTA/SN, there was again greater activity for the Atypical (M = 1.08; SD = 1.05) compared to Typical (M −0.36; SD = 1.22) actions. A paired *t* test revealed a highly significant difference (t_(32)_ = 5.97, *P* < 0.001, 95% CI = [−1.93 −0.95]).

Notably, an exploratory whole-brain analysis comparing regions more responsive to Experiment 2 mismatches (episodic and schematic violations) than Experiment 1 mismatches (only schematic violations) revealed a significant effect in the right hippocampus, supporting the relative selectivity of the hippocampal mismatch effect observed in the ROI analysis for episodic memory (*SI Appendix*, Fig. S1 and *Results, Whole-Brain Interaction*).

The commonality across Atypical actions in Experiments 1 and 2 was that they were surprising with respect to long-term semantic knowledge about the situations depicted in the videos. Therefore, common regions activated in both tasks (in particular regions of the SCN) might reflect violation of predictions based on general knowledge rather than an overarching role in all types of context-violation. Whether these regions would be activated also by context-specific, episodic memory-based violations was tested in Experiment 3.

In Experiment 3, the two sources of expectations—schematic knowledge and episodic memory—are in opposition to each other. Here, unlike the previous experiments, there were no regions more activated by Atypical than Typical actions. This suggests that familiarization with the Atypical videos rapidly diminished the fMRI response to actions that might be considered inherently surprising based on schematic knowledge. However, several regions that had been engaged by unexpected events in Experiments 1 and 2 (particularly in the dorsomedial and inferior frontal cortex) were now more engaged by Typical than Atypical actions. This reflects the regions responding to events that were unexpected based on memory for the specific videos. The only subcortical structures to also show this effect were the right amygdala and a small region of the right ventral striatum. The Network-related ANOVA revealed significant main effects for Condition (F_(1,29)_ = 19.5; *P* < 0.001; eta2[g] = 0.11) and Network (F_(1.33,38.59_) = 6.87; *P* = 0.007; eta2[g] = 0.07), and a significant Condition*Network interaction (F_(2,58)_ = 6.56; *P* = 0.003; eta2[g] = 0.03). Pairwise comparisons showed that the average response to Atypical target actions was significantly higher in the SCN (t_(29)_ = 3.56; *P* = 0.001), suggesting that these regions likely have a rather general role in processing surprising actions, regardless of the source of expectations. Additionally, the Typical actions also engaged the DMN more than did Atypical actions that matched memory for the specific context (t_(29)_ = 4.72; *P* < 0.001). Finally, expectation violation did not significantly influence activity in the MDN overall in this Experiment 3 (t_(29)_ = 1.29; *P* = 0.207). Inspection of the effect in the MDN across all three experiments suggests that in Experiment 3, activity was higher for both the Atypical and Typical actions (compared with Typical actions in Experiments 1 and 2). This is likely due to both types of actions being salient within the clips and capturing participants’ attention.

In the VTA/SN, a paired *t* test comparing Typical (M = 0.24; SD = 1.85) and Atypical (M = −0.21; SD = 1.21) actions did not reveal a statistically significant difference, t_(29)_ = 1.38, *P* = 0.178, 95% CI = [−0.22 1.12].

To summarize, we show that unexpected actions, regardless of the source of expectation violation, are processed in regions associated with generating contextually relevant inferences and resolving conflict. At the network level, Atypical, unexpected, target actions that violated general schema-based predictions engaged cognitive control networks more than did Typical, expected, actions. This is consistent with the role of SCN and MDN in resolving unexpected information by guiding contextually appropriate knowledge retrieval (45) and allocating attentional resources to interpret ambiguous situations (57, 58), respectively, suggesting that our manipulation of schema incongruence of the Atypical target actions was successful. Unexpected actions in all experiments were attention grabbing, as suggested by the increased activity in the MDN regions, important for allocating attention to salient, goal-relevant information. Those actions that also violated episodic memory-based expectations, engaged regions of the DMN, which is consistent with the DMN’s role in comparing internally generated inferences (e.g., episodic memories) with incoming information (59). Interestingly, subcortical regions associated with prediction error processing were engaged by violations of schema-based expectations, or the combination of schema- and episodic memory-based expectations, but not when expectations were purely based on episodic memory. It is therefore noteworthy that the VTA/SN showed a different pattern of activation across the three experiments compared to the hippocampus.

## Discussion

We performed three separate fMRI experiments to test the role of the hippocampus as a mismatch detector. We found that the hippocampus processes mismatches between specific episodic memories and in-the-moment experience. Critically, we show that violations of prior contextual representations based on more generalized schematic knowledge do not engage the hippocampus. These findings impose hard constraints on the information used by the hippocampus to detect mismatches. They provide direct support to theoretical models of hippocampal function that identify a limited role to processing episodic memory mismatches (7, 30). Conversely, models that have argued for a more general role for the hippocampus in comparing broader contextual representations of prior experiences to ongoing reality must be reevaluated in the light of the present results (see refs. 14 and 36).

Our study showed that Atypical sequences of actions violating long-term schematic knowledge of everyday situations did not differentially engage the hippocampus compared to Typical sequences matching schema knowledge (Experiment 1). Conversely, when participants formed episodic memories for the Typical sequences prior to scanning, viewing Atypical sequences in the scanner caused a transient hippocampal response (Experiment 2). This indicates that episodic memory-based expectations are needed to trigger hippocampal mismatch responses. Nevertheless, it remained a possibility that the additive effect of a mismatch between episodic memories and general knowledge about the situations shown exceeded a threshold for “surprise” and triggered a hippocampal response. Experiment 3 addressed this possibility, as participants prewatched all the Atypical versions of the videos before scanning. In this experiment, the contextually appropriate Typical versions of the videos caused an increased hippocampal response, since these videos mismatched episodic memories from the prewatch phase. Our findings provide strong empirical support for a family of computational models proposing a role for the hippocampus in comparing incoming information with episodic memories that are stored within the hippocampus (11, 60–62).

Our findings address a key gap in the literature since evidence in support of the hippocampus as a mismatch detector is based overwhelmingly on highly specific, recently learned, information. On the basis of such evidence, the hippocampus has been argued to have a rather general role in processing any information that mismatches expectations based on a context, or even on generalized statistical regularities learned about the world (10, 13, 14, 42). Our results strongly constrain the role of the hippocampus as a mismatch detector to situations where expectations are based on specific episodic-like representations of the event. Nevertheless, as discussed next, we do not argue that the hippocampus can only make comparisons between the current situation and a memory for an identical experience in the past—indeed such a rigid function would serve little adaptive value in most situations.

It is well established that the hippocampus is able to support flexible representations that capture the associative structure of a situation or environment and can be used to make inferences about situations that have never been experienced (63–67). How do we square the flexibility of hippocampal representations with our results? We suggest that the hippocampus only compares predictions to novel experiences that are part of a learned “cognitive map,” which may represent a physical space or a more abstract conceptual space (68). For example, a map-like representation will encode the relative locations of objects within a particular space, enabling it to detect expectation violations even if exploring the space from an entirely novel perspective (69, 70). However, when inferences must be drawn from structured mental models abstracted away from a specific cognitive map, mismatches will be detected independent of the hippocampus.

An intriguing question for future research is whether the hippocampus only signals mismatches when comparing incoming information with a specific episodic memory, or if it also signals mismatches when expectations can be very precisely predicted based on generalized knowledge. For example, while our experiences of restaurants can be highly variable and lead to rather broad expectation of what might happen in a new situation, our experiences of going through airport security might be very similar and lead to very specific expectations of what will occur (similar to expectations that can be made based on a specific episodic memory for a past experience). In the airport security example, the hippocampus might signal expectation mismatches even if these expectations are based on generalized knowledge and not with reference to any specific prior event. Alternatively, the hippocampus might only signal a mismatch if a specific episodic memory is being used to generate expectations about a new situation (e.g., my memory of passing through security at John F. Kennedy International Airport guides my expectations of what will happen at the security gates at Schiphol airport).

An alternative explanation to the prediction-mismatch account is that the hippocampal response we see reflects novelty processing more generally. In Experiments 2 and 3, the hippocampus responds to parts of the videos that have never been seen before. This could potentially reflect processing of information for which there is no preexisting representation (rather than a comparison with predictions). However, novelty detection is typically argued to involve a global match process between the input and all stored representations—irrespective of whether these representations are episodic or schematic—and therefore responses should scale with the overall degree of stimulus novelty (6, 71). Our findings are inconsistent with this; the hippocampal response in Experiment 2 was similar to Experiment 3, despite the fact that in Experiment 2 the actions were novel from the perspective of schematic knowledge, and in Experiment 3, they were not. Thus, the overall amount of novelty does not appear to be the key driver of the hippocampal response, but whether or not there is a deviation from a specific episodic memory-based representation—more in line with an associative mismatch detection account. Furthermore, our findings also argue against a broader role for the hippocampus in all forms of “contextual” novelty, as has been proposed (e.g., ref. 13).

The hippocampal BOLD response in Experiments 2 and 3 might have a number of causes. First, it might reflect the initial increase in activity of hippocampal neurons that signal the mismatch with expectations to other brain regions (11, 72). Alternatively, the detection of a mismatch in the hippocampus might lead to the activation of dopaminergic neurons in the VTA which in turn project back to the hippocampus in order to modulate learning mechanisms within the hippocampus (9, 73). For example, dopaminergic projections to the hippocampus promote long-term potentiation via activation of D1 receptors (74), which in turn has been argued to result in local increases in BOLD activity (75). However, this explanation is weakened by the fact that activity in the VTA/SN was driven by violations of schematic knowledge-based expectations, whereas the hippocampus was engaged by episodic memory-based expectation violations. Another possibility is that mismatches trigger release of acetylcholine within the hippocampus which can modulate CA1 synaptic plasticity (7, 76). Interestingly, acetylcholine can also directly affect the BOLD signal via its action as a vasodilator (77). Future research will unpick the cellular mechanisms that underpin the hippocampal mismatch response in our study.

Our results also shed light on the role of various cortical networks in processing unexpected actions. The SCN was strongly engaged when the actions were unexpected based on both schematic knowledge and the combination of schematic knowledge and episodic memory. In Experiment 3, when these sources of expectation were pitched against each other, it was the violation of episodic memories that resulted in significantly higher activity within the SCN. This network plays a role in generating inferences that are appropriate to a particular situational context (45). Our findings suggest that parts of the network additionally process context-specific expectations based on recently experienced information and not only long-term semantic knowledge (see also refs. 78 and 79). This is consistent with previous findings that regions of the SCN control context-appropriate retrieval in both episodic and semantic memory (80). The DMN showed a very similar pattern of activation to the hippocampus, suggesting a complementary role in comparing specific episodic memories with current experience. It is possible that hippocampal retrieval of the action watched before scanning triggered reinstatement of its content throughout the DMN (e.g., refs. 81 and 82). We propose that participants may have retrieved the predicted action when viewing an unexpected action, whereas this was unnecessary when observed actions aligned with prior expectations. This is consistent with predictions from a recent computational model, which suggests that episodic memory retrieval preferentially occurs when there is uncertainty about what will happen next (83).

Finally, it is notable that unexpected events were remembered differently to expected events. In Experiments 1 and 2, participants were good at recalling that *something* surprising had happened but could not necessarily remember what it was (see also ref. 84 for similar findings). This revealed itself in an increase in the number of misremembered events, which either reflected participants recalling an incorrect action or stating that the action was something strange. Interestingly, correct recall of the target actions was equivalent for both the expected and unexpected videos. This is likely to reflect two opposing effects: Surprising actions were inherently more memorable but harder to recall due to their lack of contextual relevance, while typical actions benefited from strong contextual cues (see also ref. 85). A previous study used a subset of the same video stimuli as ours, and a recognition paradigm (86). Here, the expected and unexpected actions were equivalently cued during the recognition test, and under this situation, the unexpected target actions were remembered better.

In conclusion, our findings demonstrate that the hippocampus computes mismatches between ongoing experiences and stored episodic memories but not generalized schematic knowledge. The results support theoretical models which have argued for such a limited role for the hippocampus as a comparator, but where direct experimental evidence has been lacking. Conversely, our findings constrain theories that have proposed a wider role for the hippocampus as a more general mismatch detector. Future work must clarify the degree to which hippocampal representations of structured information enable the generation of predictions in similar, yet novel, situations.

## Methods

### Participants.

There were three separate groups of healthy adult participants recruited for the three experiments. An a priori power analysis for the hippocampal contrast of Atypical vs. Typical actions suggested that 30 participants were required to detect a Cohen’s d effect size of 0.4, at a level of α = 0.05 and with a power of 0.7. A Cohen’s d effect size of 0.4 is consistent with the effect of the modulation of hippocampal response to surprising events by prediction strength reported by Long, Lee and Kuhl (23). For Experiment 1 37 participants were recruited (29 female, 8 male, range = 18 to 30 y old). One participant was excluded from any further fMRI data analysis due to issues with MRI data acquisition. In total, there were 36 participants included in the final dataset in Experiment 1 (29 female, 7 male, mean age = 21.6 y old, SD = 3.3 y). For Experiment 2, 37 participants were recruited (25 female, 12 male, range = 18 to 32 y old). Two participants were excluded from further fMRI analysis due to poor MRI data quality (excess signal dropout), and two due to not following the instructions. In total, there were 33 participants included in the final dataset in Experiment 2 (22 female, 11 male, mean age = 22 y old, SD = 3.4 y). For Experiment 3, 30 participants were recruited (25 female, 5 male, mean age = 21 y old, SD = 2.8 y, range = 18 to 29 y old). All participants had normal or corrected-to-normal vision, were right-handed, fluent English speakers. No participants were taking prescribed medication for a mental health condition. Participants were recruited via campus flyers and advertisements posted on the School of Psychology’s online participant recruitment system (Sona Systems). Informed consent was provided by all participants before the experiment, and they were given monetary compensation for participating (£10/h). All three experiments were approved by the Brighton and Sussex Medical School Research Governance and Ethics Committee.

### Stimuli.

Custom-made stop-motion video clips were used in all experiments. Thirty-four scenarios were used, and each scenario had two alternative versions resulting in 34 pairs of clips in total. The clips within each pair were identical to each other, except for one action (which we will refer to as the Target Action), which was either Typical (contextually fitting) in one version, and Atypical (incongruent with the context) in the other. Scenes including the Target Action were on average 30 s. For more information, refer to *SI Appendix*, *Stimuli*.

### Procedure.

Across the three experiments, we manipulated the typicality of sequences of actions shown within the video clips inside the scanner as well as participants’ familiarity with the video clips prior to scanning. The procedure contained three consecutive sessions—prescanning, scanning, and postscanning.

#### Prescanning session.

Before entering the fMRI scanner, participants familiarity with the specific clips was controlled. In Experiment 1, participants did not watch any video clips before entering the scanner; they were only briefed about the structure of the task that they will carry out inside the scanner. In Experiment 2, participants were familiarized with the Typical version of each video clip prior to scanning. In Experiment 3, participants were familiarized with the Atypical version of each clip prior to scanning. The familiarization included participants watching the clips in a randomized order. Then recalling each clip in as much detail as possible, in a randomized order, especially focusing on the sequence of actions carried out by the actors. Participants were cued with an image of the first frame of each clip and asked to say out loud what they remembered happening in the clips. After recalling all clips, participants watched them once again to refresh their memory. The prescanning session took 66 min to complete on average in both Experiments 2 and 3. At the end of this video familiarization phase, participants were told that inside the scanner, they will see some clips identical to the ones they had just watched and some clips that will contain a change.

#### Scanning session.

Inside the scanner, participants in all three experiments watched 34 unique clips in total—half of the clips in the Typical condition and half in the Atypical condition. Across participants, we counterbalanced which videos were seen in the Atypical and which clips were seen in the Typical condition. Each clip was seen only once during the scanning session and presentation order was randomized. In all three experiments, the scanning session lasted about an hour and followed this general trial structure: fixation-video-fixation-questionfixation-odd/even number judging task. For more information on the scanner task, refer to *SI Appendix*, *Scanning Task Supplementary Information*.

#### Postscanning session.

In all three experiments, participants were asked after scanning to recall what happened in the video clips they watched inside the scanner. Their recall was audio recorded. Participants were cued with a picture of the first frame of the scenes containing the Target Actions and were asked to recall out loud what happened in the video clip, particularly focusing on the sequence of actions the actor was performing. The recall task was self-paced, participants were given the opportunity to take a short break between recalling each clip. For more information on the postscanner task, refer to *SI Appendix*, *Postscanning Task Supplementary Information*.

### fMRI Acquisition.

All images were acquired in the Clinical Imaging Sciences Centre at the University of Sussex on a 3-T Siemens Prisma scanner with a 32-channel head coil. To minimize head movement, soft cushions were inserted into the head coil. Functional images were acquired with a gradient-echo EPI sequence with multiband acceleration factor of 3 with the following parameters (TR 1,520 ms, TE 28 ms, 75° flip angle, field of view = 208 mm × 208 mm, 72 slices with slice thickness of 2 mm and isotropic 2 mm voxels). Two SpinEcho fieldmap runs with reversed phase-encode blips in both anterior to posterior and posterior to anterior were acquired with the same parameters as the functional images. Separate field maps were acquired for each functional run. A high resolution T1-weighted image was acquired with 3-D MPRAGE sequence (in Experiments 1 and 2: TR 2,530 ms, TE 1.63 ms, 7° flip angle, field of view = 240 mm × 256 mm with slice thickness of 1 mm and 1 mm isotropic voxels; in Experiment 3: TR 2,300 ms, TE 2.19 ms, 9° flip angle, field of view = 256 mm × 256 mm with slice thickness of 1 mm and 1 mm isotropic voxels). Preprocessing steps of structural and functional data are reported in *SI Appendix*, *fMRI Preprocessing*.

### fMRI Data Analysis.

In Experiment 1, the design matrix of the GLM included five regressors. The focus of our main analyses was on transient changes in activity evoked by key moments in the task. For this reason, we included regressors for 1) the onset of the video clips; 2) the scene changes; 3) Atypical target actions; 4) Typical target actions; and 5) the full duration of the comprehension questions and following fixation cross. Note that video clips in Experiment 1 included two scenes per clip, because we also investigated BOLD activity changes at scene changes in this experiment. However, we do not report these results here. See more information about the clips in *SI Appendix*, *Stimuli*. The first four regressors had zero duration (delta functions). The onset of each target action (Atypical or Typical) corresponded to the most surprising timepoint of the Atypical version of each pair of clips. For more information on how the most surprising timepoints were chosen, refer to *SI Appendix*, *Analyses, GLM Supplementary Information*.

For Experiments 2 and 3, the design matrix of the GLM included four regressors. The regressors included the 1) video clips onsets, 2) Atypical and 3) Typical target actions, and the 4) comprehension question with the intertrial fixation crosses. All four regressors were modeled identically to Experiment 1. The only difference between the two GLMs was the absence of a scene change regressor in Experiments 2 and 3 (hence the absence of scene changes in the video clips in Experiments 2 and 3). The odd/even number judging task, the rest of the movie timepoints, and the fixation cross at the beginning of each trial were unmodeled, acting as the baseline. In all first-level models, we included six motion parameters, framewise displacement, White Matter Signal, and Cerebrospinal Fluid Signal as regressors of no interest to account for any residual noise and motion effects after motion realignment.

### Statistical Thresholding.

For whole-brain analyses, group-level testing was done using a one-sample *t* test on the functional maps generated by the first-level analysis. Whole-brain maps were cluster corrected at FWE *P* < 0.05 at voxel height defining threshold of *P* < 0.001.

### Region of Interest Analyses.

ROI masks are reported in *SI Appendix*, *Region of Interests*. In all ROI analyses, after estimating the first-level whole-brain GLMs for each participant (see GLM above), we used FSLUTILS’ fslmeants program to average the beta weights associated with the Atypical and Typical target actions from all voxels within the ROI. For the hippocampal and VTA/SN ROI analysis, we performed planned two-tailed paired *t* test comparing BOLD differences between Typical and Atypical actions in each experiment. For the hippocampal analysis, we also report complimentary Bayes Factors using two-tailed standard Cauchy prior with scale 0.707. To compare BOLD response to target actions across the Network ROIs, we conducted a repeated measures ANOVA within each experiment, with within subject factors: Condition (Typical and Atypical target actions) and Network (Semantic Control, Multiple Demand, and DMN). ANOVAs were conducted with the ez package available in R (87). In case the ANOVAs showed significant results, post hoc two-tailed paired *t* tests were conducted between conditions/networks, with Bonferroni correction applied to account for multiple comparisons.

### Behavioral Data Analysis.

Participants’ recall of all the video clips they watched inside the scanner was analyzed using logistic mixed effect models estimated with the lme4 (88) package available in R. Data analysis focused only on recall of the Target Actions (Typical or Atypical). For information on how recall was scored, refer to *SI Appendix*, *Analyses, Behavioral Data Analysis, Recall Scoring*. For further information on data exclusions in the recall analysis, refer to *SI Appendix*, *Analyses, Behavioral Data Analysis, Recall Analysis Exclusions*.

#### Remembered/forgotten target action analysis.

Our main interest was to test whether there is a difference in recalling the Target Actions correctly depending on whether the target was in the Typical or Atypical condition. Therefore, we gave a binary memory score of correct recall (1) or forgotten/incorrect (0) for each trial. We entered the binary memory score as the dependent variable and included a predictor indexing whether the target action was in the Typical (0) or Atypical (1) condition and random intercepts for participants and video clips [Recall Score ~ Condition + (1 | Participant) + (1 | Video Clip)]. To compare overall memory accuracy across experiments, we entered the binary memory score as the dependent variable and included a predictor indexing the Experiment (1,2,3) and random intercepts for participants and video clips [Recall Score ~ Experiment + (1 | Participant) + (1 | Video Clip)].

#### Memory error analysis.

We further investigated whether trials that were scored “forgotten” were simply due to the participant omitting the target action entirely from their recall or having an imperfect recall of the target action. A score of 1 was entered into the analysis for those forgotten/incorrect trials where the correct action was mentioned but the object was left out, replaced with the word “something” or replaced with another object. The rest of forgotten (omitted) trials and the remembered trials were entered into the analysis with a score of 0 (no error). We entered the binary error score as the dependent variable and included a predictor indexing whether the target action was in the Typical (0) or Atypical (1) condition and random intercepts for participants and video clips [Memory Error Score ~ Condition + (1 | Participant) + (1 | Video Clip)]. This analysis was not preregistered.

## Supplementary Material

Appendix 01 (PDF)

## Data Availability

Video clips, all code and materials; Group level contrasts between Atypical and Typical actions data have been deposited in [OSF; neurovault.org] [https://osf.io/p6z2g/ (89); https://identifiers.org/neurovault.collection:19061 (90)]. Some study data available. Our dataset consists of fMRI data from three experiments involving approximately 100 participants, with each participant undergoing around 1 h of scanning. This includes five functional runs (approximately 6 min each) and a high-resolution anatomical scan. The total dataset size is estimated to be approximately 370 GB. Due to the substantial size of the imaging data, sharing the entire dataset publicly is logistically challenging. Raw data will be made available upon request, summary nifti files of group level analysis are available on NeuroVault. All other data are included in the manuscript and/or *SI Appendix*.
