# Supplementary material for: Hippocampal mismatch signals are based on episodic memories and not schematic knowledge
Source: Proc Natl Acad Sci U S A. 2025 Aug 22;122(34):e2503535122. doi: 10.1073/pnas.2503535122 (PMC12403140; doi:10.1073/pnas.2503535122)
Supplement: Supplementary file 1 — Appendix 01 (PDF) [file pnas.2503535122.sapp.pdf]

Supporting Information for

Hippocampal Mismatch Signals are based on Episodic Memories and not Schematic Knowledge

Dominika K Varga, Petar P Raykov, Elizabeth Jefferies, Aya Ben-Yakov, Chris M Bird

Corresponding Author: Dominika Varga

Email: D.Varga@sussex.ac.uk

This PDF file includes:

Supporting text

Figure S1

Figure S2

Figure S3

Supplementary Methods

Pre-registration

For Experiment 1 and Experiment 3, the rationale, hypotheses, study design and detailed analysis plan were pre-registered (Experiment 1 Pre-registration link: https://osf.io/7g82x; Experiment 3 Pre-registration link https://osf.io/zbnt9). Experiment 2 was not pre-registered, but uses the same materials, has a similar study design and uses identical fMRI analyses as Experiment 1 and 3.

Stimuli

All video clips were custom-made, and each showed an individual person undertaking a series of actions in an everyday situation (such as doing the laundry). A range of actors were used throughout the different videos. The clips took place in different locations and depicted different scenarios. The camera maintained a fixed viewpoint throughout each scene. Thirty-four scenarios were used, and each scenario had two alternative versions resulting in 34 pairs of clips in total. 10 pairs were the same as those used in a previous study by Ben-Yakov and colleagues [1] and are available at https://osf.io/rjav4. The other 24 pairs were created to be similar to the original clips in terms of length and format. The clips within each pair were identical to each other, except for the Target Action, which was either Typical, fitting with the overall situation, or Atypical, incongruent with the context. For example, in the Typical condition of the laundry video the actor was loading a washing machine with clothes, while in the Atypical condition the actor was loading a washing machine with flowers. For each pair of clips, the timing of each action was identical, and apart from the target action, every other action was the same (e.g., entering the room, putting the washing liquid on top of the washing machine, opening the drum of the machine, closing the door after loading the machine, putting the washing liquid into the machine's drawer, etc.). For Experiment 1, which investigated surprise based on Schematic Knowledge, each video clip included two scenes: one scene with a target action (the Target Scene, average length=30 seconds) and another scene with no target action (the Non-Target Scene, average length=17 seconds). The Non-Target Scene was always related to the Target Scene but set in a different location and the person was carrying out solely context appropriate actions (such as folding clothes in a bedroom). The order of Target and Non-Target scenes were counterbalanced across the 34 clips (17 clips with Target Scene presented before, and 17 clips with the Target Scene presented after the Non-Target Scene). The reason for including Non-Target scenes in the original study of Ben-Yakov et al., [1] was to test whether surprising target actions affected memory for actions within the Non-Target scene (which it did not). We included

the Non-Target scenes in Experiment 1 in order to investigate BOLD activity changes at scene changes. However, we do not report these results here. For Experiment 2 and 3, each video clip comprised only the Target Scene. All clips of the Target Scenes are available here: https://osf.io/p6z2g/files/osfstorage

Scanning Task Supplementary Information

Due to an issue with the script that ran the stimuli in Experiment 2, 15 (out of 33) participants watched 18 clips in the Typical version and 16 in the Atypical version of the clips. All other participants (including in Experiment 1 and 3) watched 17 Typical and 17 Atypical clips.

The scanning task was carried out across 5 functional runs (Experiment 1 average length of runs = 8 minutes, Experiment 2 and Experiment 3 average length of runs = 6 minutes). Four runs contained 7 trials and 1 run contained 6 trials. A trial refers to a sequence of fixation-video-fixation-question--fixation-odd/even task. Each trial started with a red fixation cross that signalled the upcoming video (Experiment 1: Average Length = 1.30s, SD = ±0.06s; Experiment 2: Average Length = 1.29s, SD = ±0.07s; Experiment 3: Average Length = 1.35s, SD = ±0.15s). After the red fixation cross, each video started at the start of a TR. After watching a clip, participants saw a brief fixation cross (Experiment 1: Average Length = 2.22s, SD = ±0.10s; Experiment 2: Average Length = 2.22s, SD = ±0.08s; Experiment 3: Average Length = 2.19s, SD = ±0.08s) and were then asked a short Yes/No question related to the clip they just watched, which was presented for 5 seconds maximum (Experiment 1: Average RT = 2.03s, SD = ±0.37s; Experiment 2: Average RT = 0.78s, SD = ±0.22s; Experiment 3: Average RT = 0.88s, SD = ±0.24s). This was followed by a brief fixation cross again (1.5s for all trials in all experiments). Each trial ended with an Odd/Even number judging task (for 10s in all experiments). Responses to the question and the number judging task were made using a two-button response box held in the left hand (“Yes” and “Odd” with their middle finger, "No” and “Even” with their index finger). This overall task structure was consistent across all three experiments.

Participants in Experiment 1 were instructed to pay attention to the clips as they would be asked to recall them after the scanning session. The in-scanner questions assessed comprehension of the preceding video clip (e.g. half of the participants were asked “Did the actor use a washing machine?” [correct answer is “Yes”], while the other half were asked “Did the actor use a dishwasher?” [correct answer is “No”]). Participants in Experiment 2 and 3 were instructed to pay attention to the clips as they would be asked if they noticed any change to the clips compared to what they had seen prior to scanning and that they will have to recall the clips shown in the scanner after scanning. In Experiment 2, video clips in the Typical condition were

identical to those watched before scanning, but video clips in the Atypical condition replaced the previously seen Typical target action with an Atypical target action. Conversely, in Experiment 3, video clips in the Atypical condition were identical to those watched before scanning, but video clips in the Typical condition replaced the previously seen Atypical target action with a Typical target action. At the question phase, participants were asked, "Have you noticed any change in this clip?" and made a "Yes"/"No" response via the button box.

Post Scanning Task Supplementary Information

Note that in Experiment 1, the Target scenes that were cued were half of the time presented as the second scene in the video clips inside the scanner. Therefore, for half of the clips the cue did not indicate the beginning of the whole video clip, but the middle, where the scene changed. This was because our primary aim was to measure recall of the target actions; therefore, we aimed to optimise recall performance for the part of the clips that showed these actions.

fMRI Pre-processing

Preprocessing of structural and functional data were carried out using standard processing pipeline (fMRIPrep) that was developed by Esteban and colleagues to increase robustness and replicability of fMRI results [2]. Experiment 1 used fMRIPrep version 20.2.1, Experiment 2 and Experiment 3 used fMRIPrep version 21.0.0. In short, pre-processing steps involved skull-stripping the T1-weighted (T1w) image, and segmenting it into cerebrospinal fluid (CSF), white--matter (WM) and gray-matter (GM). Then the brain-extracted T1w image was registered to an MNI152 template. The BOLD functional runs were skull -stripped, head-motion parameters were estimated over the series, and slice-time correction was applied. Each BOLD timeseries was then co-registered to the native T1w image. Several confounding time-series were calculated, including framewise displacement (FD), the derivative of variance (DVARS) over frame--to-frame motion, and three region-wise global signals (from CSF, WM, and whole-brain masks). Lastly, the BOLD time-series were resampled into standard MNI152NLin2009cAsym space. Pre-processed MRI data were further analysed using MATLAB (2020b) and Statistical Parametric Mapping software (SPM12; Wellcome Trust Centre for Neuroimaging, London, UK). For whole-brain network ROI analyses, functional data were smoothed with a 6 mm FWHM kernel. For hippocampal and VTA/SN Region of Interest analyses, functional data were smoothed with a 3 mm FWHM kernel.

Region of Interests

Hippocampal ROI:

The hippocampal ROIs were taken from https://neurovault.org/collections/3731/. The main analyses included the combination of left and right “head”, “body” and “tail” ROIs into one unified bilateral hippocampal ROI. In supplementary results we also report analyses separately within the head, body and tail regions.

Additionally, we conducted analyses on the CA1, subiculum, and combined CA3-C4-Dentate Gyrus ROIs. Hippocampal subfield segmentation was performed using FreeSurfer’s automated pipeline on each participant’s T1-weighted anatomical scan. Following standard cortical reconstruction and volumetric segmentation (recon-all), hippocampal subfields were segmented using the segmentHA_T1 module, which applies a probabilistic atlas-based approach to label hippocampal subregions in T1-weighted images. Subject-specific hippocampal subfield masks were transformed to MNI152NLin2009cAsym space using the Advanced Normalization Tools (ANTs; version 2.6.1). Transformations were applied using antsApplyTransforms with the nonlinear warps produced by fMRIPrep, aligning each participant's anatomical T1-weighted image to MNI space. The MNI-normalised masks were resampled to match the resolution of each participant's preprocessed functional image (2 mm isotropic) using FSL’s flirt.

Network ROIs:

We used two control networks defined from previous studies: the semantic control network (SCN) [3] and multiple-demand network (MDN) [4]. Maps of these networks were decomposed into two non-overlapping maps containing mutually exclusive SCN regions and MDN regions [5]. A map of Default Mode Network (DMN) was taken from the 7-network parcellation from Yeo et al. [6].

Ventral tegmental Area (VTA) / substantia nigra (SN) ROI:

The VTA / SN ROI was taken from https://neurovault.org/images/786456/.

Analyses

GLM Supplementary Information

In all experiments, the onset of each target action (Atypical or Typical) corresponded to the most surprising timepoint of the Atypical version of each pair of clips. To determine the most surprising timepoints, we asked 18 independent raters to indicate the moment they found most surprising while watching the Atypical versions of each video clip. For each clip, responses that were given within the target action’s duration were averaged together to give the most surprising timepoint of that clip. These timepoints were then used as the onset for the corresponding

Atypical and Typical versions of target actions in our analysis. On average, these most surprising timepoints were 1.4 seconds after the actual onset of the target actions.

Behavioural Data Analysis

Recall scoring was identical for all experiments.

Recall Scoring

In Experiment 1, recall data from 36 participants were analysed. In Experiment 2, data from 31 participants were analysed (1 excluded due to problems at the scanning stage, 2 excluded due to not following instructions, 3 lost data). In Experiment 3, data from 30 participants were analysed. Responses were scored by one of the authors (Dominika Varga). Data scoring focused only on the target actions; other recalled details about the video were not scored. Each target action was made up of one meaningful piece of action (for example, brushing teeth) carried out with either an expected object (e.g., toothbrush) or an unexpected object (e.g., rhubarb).

Remembered / Forgotten Scoring:

Target actions were first scored as remembered (score 1) or forgotten (score 0). A target action was scored "remembered" if the participant described the action itself and the object it was carried out with. An exception to this were Typical target actions that could realistically only be performed using a specific object (such as brushing teeth with a toothbrush). Here, responses were scored as remembered even if the object was not mentioned (e.g. "the actor brushed their teeth").

Memory Error Scoring:

We further investigated whether trials that were scored "forgotten" were simply due to the participant omitting the target action entirely from their recall or having an imperfect recall of the target action. We scored two types of memory errors associated with the imperfect recall of a target action, an "Unspecified" Target memory error and a "Replaced" Target memory error. A trial was scored Unspecified Target either if the action was mentioned but the object was left out or replaced with the word "something", or, in the case of the Atypical Targets specifically, if the participant stated that the object was weird/unexpected but could not recall its specific identity. A trial was scored Replaced Target if the participant recalled the correct action but replaced the object with another object. The remainder of the forgotten trials were classified as omissions (the target action was not mentioned during recall) – i.e. were not counted as errors.

Recall Analysis Exclusions

Note that in Experiment 1 there were 10 participants who had one or more missing recall trials due to issues with the recording equipment or due to not having watched all the videos in the fMRI scanning session (percentage of trials missing across all 36 participants: M = 10%, SD = ± 23%). In Experiment 2 the 15 participants with imbalanced conditions had one target action incorrectly presented in the Typical Condition (see Scanning Session under Procedure). This incorrect trial was coded as a missing trial. 1 other participant had six trials coded as missing because they did not watch these clips inside the scanner. Missing trials were excluded from the logistic mixed effects models. There were no missing trials in Experiment 3.

Activity Time Course Analysis

We first regressed out variation due to head motion with 6 Motion Parameters, Framewise Displacement, White Matter and Cerebro Spinal Fluid signal from each functional run. Then from these filtered functional runs, we extracted and normalized (z-score) the time course from the bilateral hippocampal ROI. For each trial, we interpolated the time course to align with the true onset of the target actions. The resolution of the time course was kept as the TR resolution of 1.52 seconds. We then binned the time course around each target action TR by TR (in steps of 1.52 seconds) starting from 2 TRs before the onset of the target action and ending 10 TRs after the onset of the target action (in total 13 TRs time course window for each trial). For plotting, we averaged across each time bin for each condition within participants to get an average BOLD Signal time course for the Typical and the Atypical target actions.

Supplementary Results

Direct Comparison of Hippocampal Mismatch Effects Across Experiments 1 and 2

Although Experiments 1 and 2 differed in certain design features (e.g., video length), both included within-subject contrasts between Typical and Atypical actions, allowing us to assess whether the effect of mismatch on hippocampal responses differed meaningfully between them. Because within-subject contrasts are not influenced by global baseline shifts (e.g., due to stimulus duration), a direct comparison across experiments provides a useful test of whether the presence of episodic memory-based predictions in Experiment 2 led to significantly greater hippocampal engagement than schema-based predictions alone in Experiment 1.

To directly test for a difference in the effect of mismatches across Experiments 1 and 2, we conducted a 2 (condition: Typical Action, Atypical Action) × 2 (experiment: Experiment 1, Experiment 2) mixed-design ANOVA on hippocampal response, with condition as a within-

subjects factor and experiment as a between-subjects factor. This analysis revealed a significant interaction between condition and experiment, $F_{(1, 67)} = 14.61$, $p < .001$, eta2[g] = .084. This indicates that the effect of expectation-mismatches on hippocampal activity differed significantly between the two experiments.

As reported in the main text, expectation-mismatches modulated hippocampal responses in Experiment 2 but not in Experiment 1. The interaction confirms that this difference is statistically reliable and supports our conclusion that schematic mismatches alone are not sufficient to elicit increased hippocampal responses.

Whole-brain Interaction

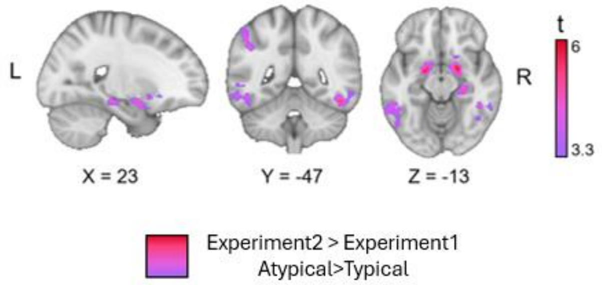

FigS1. T-map showing increased activity in the Atypical > Typical Target Actions contrast in Experiment 2 compared to Experiment 1 in several regions, including the right hippocampus. Whole brain t-maps are cluster corrected at FWE $p < .05$ at voxel height defining threshold of $p < .001$ and colour-coded to indicate the intensity of activation. The colour bar indicates the t-statistic associated with each voxel. X Y Z MNI coordinates are used for spatial reference.

Hippocampal Subregions

We examined activation separately across the hippocampal head, body, and tail to assess potential functional differentiation along the longitudinal axis. Prior research suggests that the hippocampus is not functionally homogeneous: the anterior (head) is more closely associated with emotional, schematic, and associative memory processing, while the posterior (tail) is more involved in detailed spatial and perceptual processing [7,8]. The intermediate body region may integrate features of both ends of this gradient. By analysing these subdivisions separately, we aimed to determine whether the effects of mismatches were localised to specific portions of the hippocampus or distributed uniformly along its long axis.

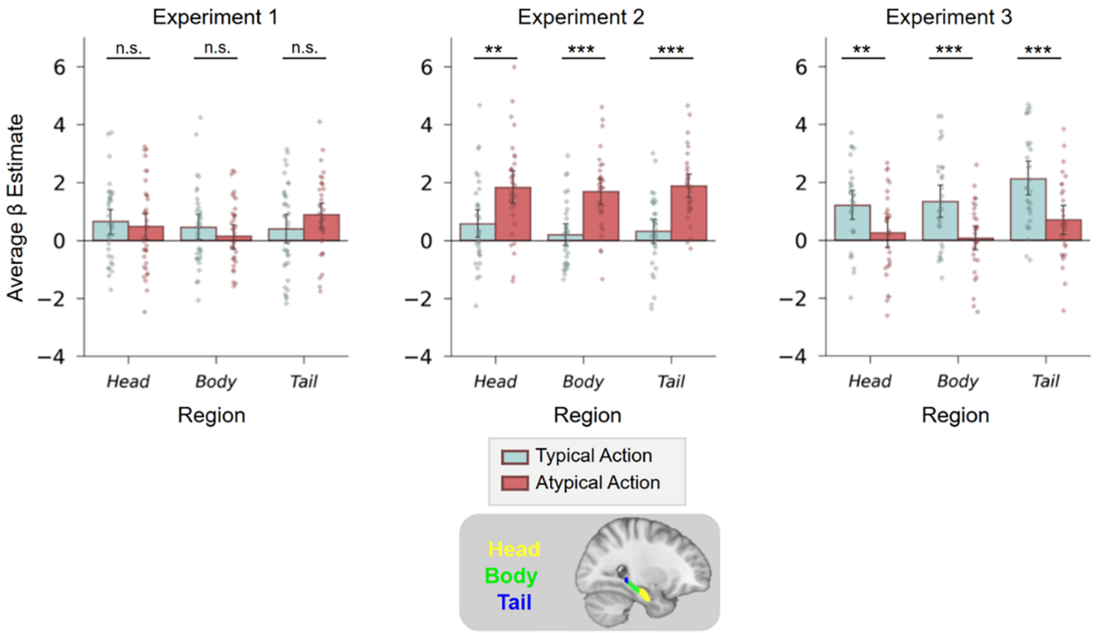

FigS2. *Uniform hippocampal subregion response to atypical vs. typical actions within experiments*. Average beta estimates for Typical and Atypical actions across hippocampal subregions (Head, Body, Tail) in each experiment. Each bar represents the mean activation per condition, with individual subject data overlaid. Error bars represent 95% confidence intervals of the mean. Pairwise comparisons were Bonferroni-corrected within each region. N.s. $p > .05$, ** $p > .001$, *** $p < .001$

Experiment 1:

A 2 × 3 repeated-measures ANOVA was conducted to assess the effects of Condition (Typical, Atypical) and Region (Head, Body, Tail) on average beta estimates. The analysis revealed no significant main effect of Condition, $F_{(1, 35)} = 0.00$, $p = .993$, eta2[g] $< .001$, indicating no overall difference in activation between Typical and Atypical actions. The main effect of Region approached significance, $F_{(1.95, 68.08)} = 2.43$, $p = .097$, eta2[g] $= .011$, suggesting a possible trend toward variation across hippocampal subregions. However, there was a significant Region × Condition interaction, $F_{(1.91, 66.91)} = 3.62$, $p = .034$, eta2[g] $= .015$, indicating that the effect of Condition varied across regions.

Bonferroni-corrected pairwise comparisons of Typical vs. Atypical actions within each Region revealed no significant differences:

Head: $t_{(35)} = 0.61$, $p = .545$; Body: $t_{(35)} = 1.00$, $p = .325$; Tail: $t_{(35)} = -1.38$, $p = .175$

In Experiment 1, hippocampal activation did not differ significantly between Typical and Atypical actions in any subregion, though the significant interaction suggests that the relationship between condition and hippocampal activation may vary subtly across subregions.

Experiment 2:

A 2 × 3 repeated-measures ANOVA was conducted to assess the effects of Condition (Typical, Atypical) and Region (Head, Body, Tail) on average beta estimates. The analysis revealed a significant main effect of Condition, $F_{(1, 32)} = 37.92$, $p < .001$, eta2[g] = .231, indicating a strong difference in average activation between the two conditions. However, the main effect of Region was not significant, $F_{(1.71, 54.74)} = 0.93$, $p = .389$, eta2[g] = .007, suggesting similar BOLD signal levels across hippocampal subregions. Additionally, the Region × Condition interaction was not significant, $F_{(1.80, 57.54)} = 0.44$, $p = .624$, indicating that the condition effect was consistent across all subregions.

Bonferroni-corrected pairwise comparisons of Typical vs. Atypical actions within each Region revealed significant differences across all subregions, with Atypical actions consistently producing higher average beta estimates:

Head: $t_{(32)} = -3.52$, $p = .001$; Body: $t_{(32)} = -5.46$, $p < .001$; Tail: $t_{(32)} = -5.76$, $p < .001$

Experiment 3:

A 2 × 3 repeated-measures ANOVA was conducted to examine the effects of Condition (Typical, Atypical) and Region (Head, Body, Tail) on average beta estimates. The analysis revealed a significant main effect of Region, $F_{(1.66, 48.23)} = 5.64$, $p = .009$, eta2[g] = .049, indicating that average signal significantly varied across hippocampal subregions. There was also a significant main effect of Condition, $F_{(1, 29)} = 27.54$, $p < .001$, eta2[g] = .152, with higher signal in Typical compared to Atypical actions. The Region × Condition interaction was not significant, $F_{(1.79, 51.87)} = 1.21$, $p = .304$, suggesting the effect of Condition was consistent across Regions.

Bonferroni-corrected pairwise comparisons of Typical vs. Atypical actions within each Region showed significant differences across all subregions, with Typical actions consistently producing higher average beta estimates:

Head: $t_{(29)} = 3.37$, $p = .002$; Body: $t_{(29)} = 4.90$, $p < .001$; Tail: $t_{(29)} = 4.42$, $p <.001$

Overall, these findings confirm that actions surprising based on prior episodic memories consistently produced higher hippocampal activation compared to unsurprising actions along the hippocampal anterior-posterior axis.

Hippocampal Subfields

To further examine the specificity of hippocampal subfield responses to surprising actions, we focused additional analyses on CA1 and the subiculum. CA1 is considered a major convergence zone for hippocampal processing, involved in comparing incoming input with stored representations [9, 10]. Additionally, the subiculum may also contribute to novelty-related processing, particularly in encoding contextual change and relaying prediction error signals to broader cortical networks [11,12,13.] To determine whether observed effects are specific to CA1 and subiculum or more broadly distributed within the hippocampus, we created a comparison ROI by combining CA3, CA4, and the dentate gyrus (DG). These regions are functionally interconnected as part of the hippocampal trisynaptic circuit, a canonical pathway in which input from the entorhinal cortex passes through the dentate gyrus (including the hilus/CA4) to CA3 and then to CA1 [14,15]. Importantly, the combined CA3–CA4–DG ROI yields a volume comparable to CA1, allowing for a size-matched comparison that helps control for potential confounds related to ROI extent or signal-to-noise differences.

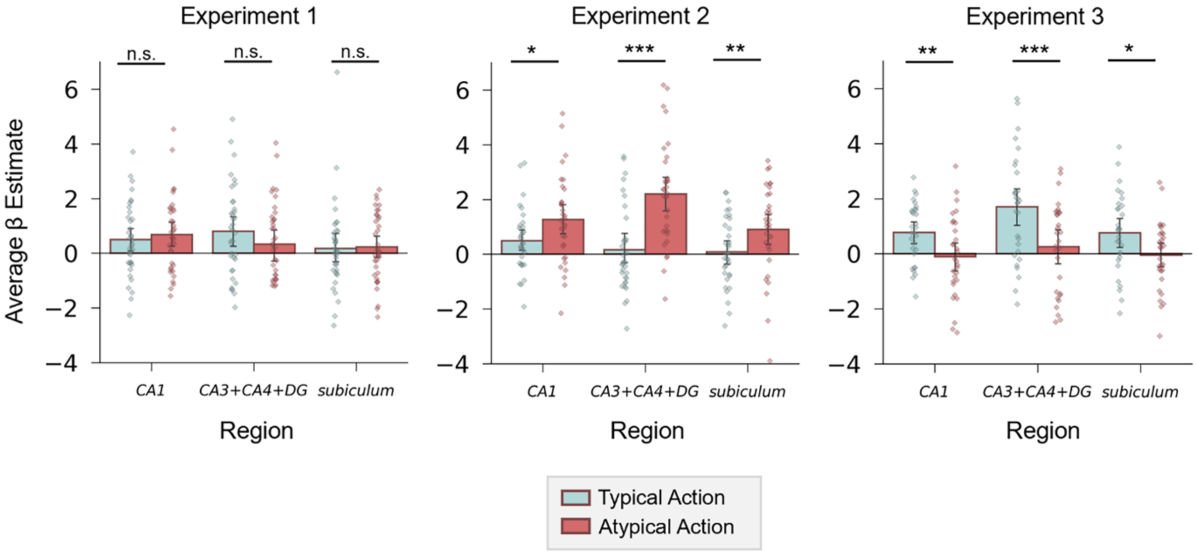

FigS3. *Uniform hippocampal subfield response to atypical vs. typical actions within experiments*. Average beta estimates for Typical and Atypical actions across hippocampal subfields (CA1, CA3+CA4+DG, Subiculum) in each experiment. Each bar represents the mean activation per condition, with individual subject data overlaid. Error bars represent 95% confidence intervals of the mean. Pairwise comparisons were Bonferroni-corrected within each region. n.s. $p > .05$, * $p > .01$, ** $p > .001$, *** $p < .001$

Experiment 1:

A repeated-measures ANOVA was conducted on average beta estimates with Region (CA1, CA3+CA4+DG, Subiculum) and Condition (Typical, Atypical) as within-subjects factors. A

significant main effect of Region was observed, $F_{(1.93, 67.52)} = 3.29$, $p = .045$, eta2[g] = .014, indicating that average level of response differed across hippocampal subregions. The main effect of Condition was not significant, $F_{(1, 35)} = 0.09$, $p = .762$, eta2[g] < .001, suggesting that typical and atypical stimuli elicited comparable responses overall.
Importantly, there was no significant Region × Condition interaction, $F_{(1.97, 69.03)} = 2.39$, $p = .100$, eta2[g] = .009, indicating that the effect of Condition did not differ significantly across regions. Bonferroni-corrected comparisons of Condition within each Region further confirmed the absence of significant differences:

CA1: $t_{(35)} = -0.64$, $p = .525$; CA3/CA4/DG: $t_{(35)} = 1.28$, $p = .208$; Subiculum: $t_{(35)} = -0.17$, $p = .865$

These findings suggest that hippocampal response varied by region but was not significantly modulated by the unexpectedness of actions in any hippocampal subregion in Experiment 1.

Experiment 2:

A repeated-measures ANOVA was conducted on average beta estimates with Region (CA1, CA3+CA4+DG, Subiculum) and Condition (Typical, Atypical) as within-subjects factors. There were significant main effects of Region ($F_{(1.89, 60.63)} = 7.34$, $p = .002$, eta2[g] = .033) and Condition ($F_{(1, 32)} = 18.43$, $p < .001$, eta2[g] = .138). A significant Region × Condition interaction was also observed ($F_{(1.88, 60.23)} = 6.61$, $p = .003$, eta2[g] = .036).
To unpack the interaction, pairwise comparisons of Condition (Typical vs. Atypical) were conducted within each Region, with Bonferroni correction for multiple comparisons. In all regions, average signal was significantly higher for the Atypical condition:

CA1: $t_{(32)} = -2.33$, $p = .027$; CA3/CA4/DG: $t_{(32)} = -4.61$, $p < .001$; Subiculum: $t_{(32)} = -2.77$, $p = .009$

These findings indicate that atypical actions elicited reliably increased responses across hippocampal subfields when both schematic and episodic expectations were violated, with the strongest effect observed in CA3/CA4/DG in Experiment 2.

Experiment 3:

A repeated-measures ANOVA was conducted on average beta estimates with Region (CA1, CA3+CA4+DG, Subiculum) and Condition (Typical, Atypical) as within-subjects factors.There were significant main effects of Region, $F_{(1.97, 57.09)} = 5.75$, $p = .005$, eta2[g] = .040, and Condition, $F_{(1, 29)} = 20.32$, $p < .001$, eta2[g] = .113, with higher activity in response to typical

compared to atypical actions. There was no significant Region × Condition interaction, $F_{(1.94, 56.32)} = 1.58$, $p = .215$, eta2[g] = .009, indicating that the effect of Condition did not differ significantly across subfields.

Bonferroni-corrected pairwise comparisons of Condition (Typical vs. Atypical) within each Region showed significantly higher activity in response to typical compared to atypical actions in all three subfields:

CA1: $t_{(29)} = 3.45$, $p = .002$; CA3/CA4/DG: $t_{(29)} = 3.87$, $p < .001$; Subiculum: $t_{(29)} = 2.43$, $p = .021$

These results suggest that typical actions consistently evoked increased responses compared to atypical actions across all examined hippocampal subfields when those actions violated episodic memories of what previously happened in the clips.

Together, the pattern of findings highlights the hippocampus's general sensitivity to mismatches when episodic memories are involved in the comparison.

SI References

1. Ben-Yakov, A., Smith, V. & Henson, R. (2022) The limited reach of surprise: Evidence against effects of surprise on memory for preceding elements of an event. *Psychon Bull Rev 29*, 1053–1064.
2. Esteban, O. *et al.* (2018) fMRIPrep: a robust preprocessing pipeline for functional MRI. *Nature Methods 2018 16:1 16*, 111–116.
3. Jackson, R. L. (2021) The neural correlates of semantic control revisited. *Neuroimage 224*, 117444.
4. Fedorenko, E., Duncan, J. & Kanwisher, N. (2013) Broad domain generality in focal regions of frontal and parietal cortex. *Proc Natl Acad Sci U S A 110*, 16616–16621.
5. Gao, Z. *et al.* (2021) Distinct and common neural coding of semantic and non-semantic control demands. *Neuroimage 236*,.
6. Yeo, B. T. *et al.* (2011) The organization of the human cerebral cortex estimated by intrinsic functional connectivity. *J Neurophysiol 106*, 1125–1165.
7. Poppenk, J., Evensmoen, H. R., Moscovitch, M., & Nadel, L. (2013). Long-axis specialization of the human hippocampus. *Trends in cognitive sciences*, *17*(5), 230-240.
8. Strange, B. A., Witter, M. P., Lein, E. S., & Moser, E. I. (2014). Functional organization of the hippocampal longitudinal axis. *Nature reviews neuroscience*, *15*(10), 655-669.
9. Lisman, J. E., & Grace, A. A. (2005). The hippocampal-VTA loop: controlling the entry of information into long-term memory. *Neuron*, *46*(5), 703-713.
10. Duncan, K., Ketz, N., Inati, S. J. & Davachi, L. (2012) Evidence for area CA1 as a match/mismatch detector: A high-resolution fMRI study of the human hippocampus. *Hippocampus 22*, 389–398.
11. Naber, P. A., Witter, M. P., & Lopes da Silva, F. H. (2000). Networks of the Hippocampal Memory System of the Rat: The Pivotal Role of the Subiculum a. *Annals of the New York Academy of Sciences*, *911*(1), 392-403.
12. Lever, C., Burton, S., Jeewajee, A., Wills, T. J., Cacucci, F., Burgess, N., & O'Keefe, J. (2010). Environmental novelty elicits a later theta phase of firing in CA1 but not subiculum. *Hippocampus*, *20*(2), 229-234.

13. Aggleton, J. P., & Christiansen, K. (2015). The subiculum: the heart of the extended hippocampal system. *Progress in brain research*, *219*, 65-82.

14. Rolls, E. T. (2013). The mechanisms for pattern completion and pattern separation in the hippocampus. *Frontiers in systems neuroscience*, *7*, 74.

15. Amaral, D. & Lavenex, P. (2007) Hippocampal neuroanatomy.
